# Ultrasound detection of septic jugular vein thrombosis

**DOI:** 10.1186/2036-7902-4-S1-A3

**Published:** 2012-12-18

**Authors:** Nina Kobilica, A Bergauer, V Flis

**Affiliations:** 1 Surgical Clinic, Department of Vascular Surgery, University Medical Center Maribor, Ljubljanska 5, Slovenia

## 

The two leading causes of IJVT are iatrogenic trauma secondary to jugular vein catheterization, and repeated IV injections by drug users. Lemierre syndrome is a complex and unusual clinical entity, characterized by septic thrombophlebitis of internal jugular vein. Lemierre syndrome was thought to be a rare and forgotten disease with suggested incidence of approximately one per million. However, an increase in frequency over the past years has been suggested due to changes in antibiotic usage. Unfortunately, wide spread antibiotic usage has also changed clinical picture of Lemierre syndrome and it is often difficult to recognize this unusual ilness in the Emergency Department (ED). Systemic septic complications may range from deep neck infection over septic arthritis to brain infections. Every organ system may be involved. Delays in diagnosis ranging up to 11 days after admission have been reported. When recognized and treated in early phase patients recover completely but other vise condition may be lethal. In emergency settings accurate and prompt diagnosis is crucial in satisfactory patient management. Diagnosis of Lemierre's syndrome is simple with Doppler ultrasonography but it mostly requires a high degree of clinical suspicion. It has been suggested that bedside ultrasound of the internal jugular vein in ED before other radiologic imaging, may lead to rapid diagnosis and treatment of Lemierre syndrome. In last two years we treated five patients with Lemierre’s syndrome in our department. In one case young woman died because of sepsis and multiorgan failure due to delayed diagnosis. Rapid ultrasound examination of neck veins is discussed as a part of ED evaluation of patients.

